# Integrated Models of Care for People Living with Hepatitis C Virus and a Substance Use Disorder: Protocol for a Systematic Review

**DOI:** 10.2196/resprot.9532

**Published:** 2018-05-09

**Authors:** Lianping Ti, Stephanie Parent, María Eugenia Socías

**Affiliations:** ^1^ British Columbia Centre for Excellence in HIV/AIDS Vancouver, BC Canada; ^2^ Department of Medicine University of British Columbia Vancouver, BC Canada; ^3^ British Columbia Centre for Substance Use Vancouver, BC Canada

**Keywords:** hepatitis C virus, substance use disorder, integrated care, protocol, systematic review

## Abstract

**Background:**

People living with a substance use disorder (SUD) are a key population within the hepatitis C virus (HCV) epidemic. While integrated and community-based models of care have shown positive outcomes among this population, the literature has been primarily focused on the HIV context. This paper outlines a systematic review protocol on the impact of various integrated models of care, which includes HCV and SUD services, on various treatment, and health-related outcomes among this population.

**Objective:**

The objective of this review is to determine the impact of integrated models of care on HCV and addiction treatment and health-related outcomes for adults living with HCV and an SUD.

**Methods:**

We will search 5 databases, article reference lists, and abstracts from relevant conferences that investigate the impact of integrated models of care on treatment and health-related outcomes among people living with HCV and an SUD. Database searches will be conducted and titles, abstracts, and the full-text of the relevant studies will be independently reviewed in separate stages. The methodological quality of included studies will be assessed using a validated tool. Data from included articles will be extracted using a standardized form and synthesized in a narrative account.

**Results:**

For this project, we have received funding from the Canadian Institute of Health Research. To date, we have completed the search strategy, reviewed the titles, abstracts, and full-texts. Grading the selected studies and qualitative synthesis of the results are currently under way, and we expect the final results to be submitted for publication in the fall of 2018.

**Conclusions:**

The systematic review will describe different integrated models of care that could be effective in improving the health and well-being of people living with HCV and an SUD. Results of this review could also identify quality improvement strategies to minimize the health and cost burden imposed on patients, healthcare professionals, and the healthcare system.

**Trial Registration:**

PROSPERO CRD42017078445; https://www.crd.york.ac.uk/prospero/display_record.php?RecordID=78445 (Archived by WebCite at http://www.webcitation.org/6z4YnkE9G)

## Introduction

People living with a substance use disorder (SUD) are a key population within the hepatitis C virus (HCV) epidemic. Specifically, the estimated global HCV prevalence among people who inject drugs (PWID) is 67%, or approximately 10 million individuals, with incidence rates ranging from 5% to 45% per year [1]. PWID constitute approximately 14% of the 71 million people infected with HCV globally [2]. Due to similar transmission routes, there is overrepresentation of PWID among individuals who are co-infected with HCV and HIV, with over half of the 2.3 million globally co-infected individuals being among PWID [3,4]. PWID are not only at greater risk of HCV infection (and re-infection), but also at increased risk of disease progression, mortality, and onward HCV transmission due to various socio-structural and environmental exposures, including poor access to health care services, socioeconomic marginalization, and the persistence of law enforcement-based responses to illicit drug use [1,5-7]. While HCV is mainly transmitted via injection drug use, non-injection drug use has also been associated with HCV infection. For example, there is some evidence of HCV transmission via crack pipe sharing [8], and among men who have sex with men who use non-intravenous drugs [9,10]. HCV prevalence is higher in non-injecting drug users than in nondrug users. Moreover, many PWID are polysubstance users with non-injection drugs [11].

Despite the advent of safe and highly efficacious direct-acting antiviral–based therapy, resulting in the possibility of controlling the HCV epidemic, concerns regarding treatment access, adherence, and potential reinfection, particularly among marginalized populations such as PWID, remain [5,6]. Indeed, access and uptake of HCV treatment has been consistently low among PWID, with studies indicating that only 1%-6% of this population has ever been treated with interferon-based therapies [12-14]. The reluctance of physicians to treat HCV infection in PWID is likely due to the stigma and discrimination that continue to persist among this population [15]. Low treatment rates among this population may also reflect PWIDs’ fear of treatment, competing priorities (eg, active illicit drug use), as well as general barriers to accessing healthcare [16,17]. Moreover, in some jurisdictions, the high cost of direct-acting antivirals (DAA) treatment is only covered provided that the patient meets specific criteria (eg, a fibrosis score of 2 or more) [18]. As such, the high cost of DAA may also constitute a barrier to treatment for PWID who do not meet these criteria, or who live in settings where DAA treatment is not covered. Nevertheless, recent studies have shown that, when appropriately supported, PWID are able to achieve similar rates of sustained virologic response compared to the general population [19,20].

Prior reviews have explored integrated models of care in the context of HIV, and these have been shown to have a significant beneficial impact on the treatment and health outcomes of PWID, particularly when coupled with SUD services [21]. Similarly, emerging studies have suggested the importance of opioid agonist therapy (OAT) and mental health services on reducing the risk of HCV reinfection among this population [19,22-24]. However, there has been no explicit systematic review conducted on the impact of HCV and SUD integrated programs and services on HCV, and addiction treatment and health outcomes among individuals living with these comorbid diseases. This review will address an important gap in the literature by comprehensively assessing the available literature to provide insight into effective and efficient models of care for people living with HCV and SUD.

## Methods

The current protocol has been registered in the PROSPERO CRD42017078445 and conforms to the Preferred Reporting Items for Systematic Reviews and Meta-Analyses Protocols (PRISMA-P) checklist (Multimedia Appendix 1). We will also adhere to the PRISMA guidelines for the development of this systematic review report [25].

### Research Question

The proposed systematic review will synthesize the existing literature on HCV and SUD integrated models of care to date. Specifically, this review aims to answer the following research question: what is the impact of integrated models of care on HCV and addiction treatment outcomes (eg, HCV treatment adherence, OAT uptake), health-related outcomes (eg, HCV clearance), and cost-related outcomes (eg, long-term cost effectiveness) for adults living with HCV and an SUD?

### Eligibility

Original quantitative and qualitative research studies that reported on integrated care models for adults living with HCV and an SUD will be included. For the present study, substance use will be defined as any use of alcohol, illicit use or misuse of opioids (eg, heroin, prescription opioids, methadone, morphine, oxycodone), or stimulants (eg, cocaine, methamphetamine). We will include studies that describe service integration interventions at varying degrees of integration. Some examples may include HCV screening or treatment within OAT clinics or multidisciplinary care addressing medical, psychological, social, and addiction-related needs.

Commentaries, letters to editors, editorials, and other types of opinion pieces will be excluded. Literature reviews will also be excluded; however, we will conduct back referencing to ensure that all relevant studies from the literature review are captured. The search will not be restricted to setting. Moreover, we will not exclude studies based on study design, but the degree of bias will be noted in interpreting the findings. The search will be restricted to publications in English, French, and Spanish.

### Information Sources and Search Strategy

A comprehensive search strategy to identify documents that met the eligibility criteria will be conducted. Specifically, the databases MEDLINE, EMBASE, CINAHL, PsycINFO, and Web of Science will be searched with no date restrictions in order to acknowledge changes in care over time. However, we will ensure that the study period is included in our analysis. In addition, we will search ClinicalTrials.gov to ensure that we capture studies that have not yet been published. Consistent with similar reviews [21], search terms will include those that are related to three themes, namely HCV, substance use, and integrated healthcare services, and these terms will be mapped to database-specific medical subject headings and controlled vocabulary terms when available (Multimedia Appendix 2).

To maximize the number of included studies, hand-searching of full-text scientific conference proceedings from HCV- and substance use conferences (eg, American Association for the Study of Liver Diseases, European Association for the Study of the Liver, Conference on Retroviruses and Opportunistic Infections, International AIDS Society Conference) will be conducted and will be restricted to be within the previous two years. We restricted the conference abstract search to the past two years in order to limit duplication with older abstracts that may have been published as academic articles. We will also search reference lists of research articles and reviews by hand to identify relevant articles not otherwise captured. These search methods have been developed in consultation with a medical reference librarian with expertise in systematic reviews and population and public health at the University of British Columbia (U. Ellis, personal communication, June 12, 2017) and will be executed by an author experienced in conducting systematic reviews.

### Study Records

Database searches will be conducted and the abstracts and full-text articles from the search strategy will be imported into Endnote X7. After removing duplicates, the titles, abstracts, and full text of articles will be independently reviewed in three separate stages by two research team members. The first stage will consist of reviewing the titles. The second stage will consist of reviewing the abstracts. The third stage will be the review of the full text of the articles. At each review stage, studies clearly not meeting the inclusion criteria will be excluded from further review and the reason for exclusion will be recorded. Any disagreements between the two investigators will be resolved by discussion with a third investigator.

### Risk of Bias in Individual Studies

The methodological quality, including risk of bias, of included quantitative research studies will be assessed using a modified version of the Downs and Black checklist for the reporting of healthcare studies, which has been shown to be a valid and reliable tool [26,27]. Higher scores out of a total score of 18 represent higher overall methodological quality. Qualitative studies will be assessed using the Critical Appraisal Skills Programme tool [28], a widely used tool recommended by some journals [29]. Each study will be scored by one investigator and verified independently by a second investigator. Where there were disagreements in scoring, this will be resolved by discussion with a third investigator.

### Data Synthesis

Following PRISMA guidelines, a flow chart of the selection process will be produced. Additionally, data from included studies will be extracted using a standardized form developed to capture study characteristics and main findings and summarized in a table, including information on: study characteristics (eg, study setting, study design, study period, and study population), participant characteristics (eg, age, sex or gender), study objectives, integrated service intervention type and type of facility, and main study findings. Findings from the included studies will then be synthesized in a narrative account that addresses the objectives of this systematic review.

## Results

We have received funding from the Canadian Institute of Health Research, allowing the commencement of the project. To date, we have completed the search strategy. We obtained 1711 records after duplicates were removed. After screening the titles and abstracts, 153 full-texts were reviewed. Of those, 57 were excluded and 96 will be included in the qualitative synthesis. After hand-searching conferences abstracts, 36 were selected for inclusion. We are currently in the process of grading the selected full-texts, and qualitative synthesis of the findings is currently under way. We expect the final results to be submitted for publication in the fall of 2018.

## Discussion

To our knowledge, this systematic review will be the first to synthesize the available evidence on the integration of HCV and substance use services on treatment, health, and cost-related outcomes. Identification and implementation or adaption of different integrated models of care to improve the health and well-being of people living with HCV and an SUD may have a significant impact on reducing the negative health and social consequences associated with these comorbid diseases, as well as healthcare utilization costs, and resource burden on the healthcare system. The results of this systematic review may guide future research in this area and contribute to the development of evidence-based policies and programs for the treatment and care of people living with HCV and an SUD.

We plan to implement a comprehensive end-of-project knowledge translation strategy to ensure that the findings of this research are accessible to key stakeholders. Specifically, we will present the results of this review at relevant HCV and substance use meetings nationally and internationally, as well as publish in an open access peer-reviewed journal in an effort to increase access for appropriate scientific, clinical, and public audiences. Lastly, we plan to collaborate with relevant clinical programs and community organizations to ensure the timely and effective application of the research findings. Specifically, we will organize plain language presentations of our research findings with time for discussion and feedback, we will attend meetings and planning discussions with health authorities and health administrators, and we will reach out to policy-makers via briefing notes or other venues.

There are several limitations that should be noted. The expected heterogeneity of the literature on integrated models of care may affect our ability to draw clear conclusions from the literature. Additionally, we recognize that the selection and qualitative synthesis of the eligible studies is a subjective process; however, we will seek to minimize this limitation by duplicating our search and utilizing two reviewers to conduct the screening and quality assessment processes independently. As with all reviews, it is possible that some eligible studies may be missed in our search strategy. To minimize this limitation, we have kept our search strategy relatively broad and have sought input from an experienced librarian. Lastly, there may be a publication bias observed as a general limitation of systematic reviews. We will try to minimize this issue by searching databases for unpublished studies (eg, ClinicalTrials.gov).

In sum, this systematic review will synthesize the available evidence on the integration of HCV and substance use services and its impact on various outcomes, including health- and cost-related outcomes. It is expected that the findings from this review will provide evidence towards the effective delivery of healthcare programs and services for people living with HCV and an SUD.

## Appendix

Multimedia Appendix 1 Preferred Reporting Items for Systematic Reviews and Meta-Analyses Protocols (PRISMA-P) checklist.

Multimedia Appendix 2 Search Strategy in OVID MEDLINE.

## Abbreviations

DAA: direct-acting antivirals
HCV: hepatitis C
OAT: opioid agonist therapy
PRISMA: Preferred Reporting Items for Systematic Reviews and Meta-Analyses Protocols
PRISMA: people who inject drugs
SUD: substance use disorder

## References

[1] Nelson PK, Mathers BM, Cowie B, Hagan H, Des JD, Horyniak D, Degenhardt L (2011). Global epidemiology of hepatitis B and hepatitis C in people who inject drugs: results of systematic reviews. Lancet.

[2] (2017). World Health Organization.

[3] Platt L, Easterbrook P, Gower E, McDonald B, Sabin K, McGowan C, Yanny I, Razavi H, Vickerman P (2016). Prevalence and burden of HCV co-infection in people living with HIV: a global systematic review and meta-analysis. Lancet Infect Dis.

[4] (2017). World Health Organization.

[5] Backmund M, Meyer K, Von ZM, Eichenlaub D (2001). Treatment of hepatitis C infection in injection drug users. Hepatology.

[6] Grady BP, Schinkel J, Thomas XV, Dalgard O (2013). Hepatitis C virus reinfection following treatment among people who use drugs. Clin Infect Dis.

[7] Hagan H, Thiede H, Des JDC (2004). Hepatitis C virus infection among injection drug users: survival analysis of time to seroconversion. Epidemiology.

[8] Macías J, Palacios RB, Claro E, Vargas J, Vergara S, Mira JA, Merchante N, Corzo JE, Pineda JA (2008). High prevalence of hepatitis C virus infection among noninjecting drug users: association with sharing the inhalation implements of crack. Liver Int.

[9] Götz HM, van DG, Niesters HG, den HJG, Thio HB, de ZO (2005). A cluster of acute hepatitis C virus infection among men who have sex with men--results from contact tracing and public health implications. AIDS.

[10] Scheinmann R, Hagan H, Lelutiu-Weinberger C, Stern R, Des JDC, Flom PL, Strauss S (2007). Non-injection drug use and Hepatitis C Virus: a systematic review. Drug Alcohol Depend.

[11] Hayden A, Hayashi K, Dong H, Milloy M, Kerr T, Montaner JSG, Wood E (2014). The impact of drug use patterns on mortality among polysubstance users in a Canadian setting: a prospective cohort study. BMC Public Health.

[12] Mehta SH, Genberg BL, Astemborski J, Kavasery R, Kirk GD, Vlahov D, Strathdee SA, Thomas DL (2008). Limited uptake of hepatitis C treatment among injection drug users. J Community Health.

[13] Iversen J, Grebely J, Topp L, Wand H, Dore G, Maher L (2014). Uptake of hepatitis C treatment among people who inject drugs attending Needle and Syringe Programs in Australia, 1999-2011. J Viral Hepat.

[14] Grebely J, Raffa JD, Lai C, Kerr T, Fischer B, Krajden M, Dore GJ, Tyndall MW (2011). Impact of hepatitis C virus infection on all-cause and liver-related mortality in a large community-based cohort of inner city residents. J Viral Hepat.

[15] Habib SE, Adorjany LV (2016). Hepatitis C and injecting drug use: The realities of stigmatisation and discrimination. Health Education Journal.

[16] Edlin BR, Kresina TF, Raymond DB, Carden MR, Gourevitch MN, Rich JD, Cheever LW, Cargill VA (2005). Overcoming barriers to prevention, care, and treatment of hepatitis C in illicit drug users. Clin Infect Dis.

[17] Jones L, Atkinson A, Bates G, McCoy E, Porcellato L, Beynon C, McVeigh J, Bellis MA (2014). Views and experiences of hepatitis C testing and diagnosis among people who inject drugs: systematic review of qualitative research. Int J Drug Policy.

[18] (2017). British Columbia Ministry of Health.

[19] Dimova RB, Zeremski M, Jacobson IM, Hagan H, Des JDC, Talal AH (2013). Determinants of hepatitis C virus treatment completion and efficacy in drug users assessed by meta-analysis. Clin Infect Dis.

[20] Aspinall EJ, Corson S, Doyle JS, Grebely J, Hutchinson SJ, Dore GJ, Goldberg DJ, Hellard ME (2013). Treatment of hepatitis C virus infection among people who are actively injecting drugs: a systematic review and meta-analysis. Clin Infect Dis.

[21] Haldane V, Cervero-Liceras F, Chuah FL, Ong SE, Murphy G, Sigfrid L, Watt N, Balabanova D, Hogarth S, Maimaris W, Buse K, Piot P, McKee M, Perel P, Legido-Quigley H (2017). Integrating HIV and substance use services: a systematic review. J Int AIDS Soc.

[22] Perlman DC, Jordan AE, Uuskula A, Huong DT, Masson CL, Schackman BR, Des JDC (2015). An international perspective on using opioid substitution treatment to improve hepatitis C prevention and care for people who inject drugs: Structural barriers and public health potential. Int J Drug Policy.

[23] Dore GJ, Altice F, Litwin AH, Dalgard O, Gane EJ, Shibolet O, Luetkemeyer A, Nahass R, Peng C, Conway B, Grebely J, Howe AYM, Gendrano IN, Chen E, Huang H, Dutko FJ, Nickle DC, Nguyen B, Wahl J, Barr E, Robertson MN, Platt HL, C-EDGE CO-STAR Study Group (2016). Elbasvir-Grazoprevir to Treat Hepatitis C Virus Infection in Persons Receiving Opioid Agonist Therapy: A Randomized Trial. Ann Intern Med.

[24] Platt L, Minozzi S, Reed J, Vickerman P, Hagan H, French C, Jordan A, Degenhardt L, Hope V, Hutchinson S, Maher L, Palmateer N, Taylor A, Bruneau J, Hickman M (2017). Needle syringe programmes and opioid substitution therapy for preventing hepatitis C transmission in people who inject drugs. Cochrane Database Syst Rev.

[25] Moher D, Liberati A, Tetzlaff J, Altman DG (2010). Preferred reporting items for systematic reviews and meta-analyses: the PRISMA statement. Int J Surg.

[26] Downs SH, Black N (1998). The feasibility of creating a checklist for the assessment of the methodological quality both of randomised and non-randomised studies of health care interventions. J Epidemiol Community Health.

[27] Marshall BDL, Werb D (2010). Health outcomes associated with methamphetamine use among young people: a systematic review. Addiction.

[28] Critical Appraisal Skills Programme.

[29] Neale J, West R (2015). Guidance for reporting qualitative manuscripts. Addiction.

