# Supplementary material for: Integrated Models of Care for People Living with Hepatitis C Virus and a Substance Use Disorder: Protocol for a Systematic Review
Source: JMIR Res Protoc. 2018 May 9;7(5):e122. doi: 10.2196/resprot.9532 (PMC5966653; doi:10.2196/resprot.9532)
Supplement: Multimedia Appendix 2 [file resprot_v7i5e122_app2.pdf]

Additional File 2

Search strategy in OVID Medline

Number	Search statement	Number of results
1	exp hepatitis c/ or hepatitis c.mp or HCV.mp	84484
2	(substance-related disorder* or substance related disorder*).mp or exp substance-related disorders/ or illicit drug*.mp or drug use*.mp or PWID.mp or IDU.mp or PWUD.mp or opiate*.mp or heroin.mp or alcohol*.mp or cocaine.mp or tobacco.mp	715784
3	"Delivery of Health Care, Integrated"/	10559
4	((vertical or horizontal or integrat* or coordinat* or co-ordinat* or link*) and (program* or care or service* or system*))	535833
5	3 or 4	535833
6	1 and 2 and 5	557
